# Therapeutic Plasma Exchange in Intensive Care Units—A 5-Year Multicenter Retrospective Study

**DOI:** 10.3390/life15091406

**Published:** 2025-09-06

**Authors:** Daria Syguła, Paulina Kluszczyk, Wiktor Wagner, Michał Krawiec, Szymon Bialka, Ewa Trejnowska, Grzegorz Brozek, Magdalena Latos, Paweł Dubik, Piotr Palaczynski, Piotr Knapik, Szymon Skoczyński

**Affiliations:** 1 Student Scientific Society, Department of Lung Diseases and Tuberculosis, Faculty of Medical Sciences in Zabrze, Medical University of Silesia, Medyków 14 St., 40-752 Katowice, Poland; 2 Student Scientific Society, Department of Anaesthesiology and Critical Care, School of Medicine with Division of Dentistry in Zabrze, Medical University of Silesia, Plac Traugutta 2 St., 41-800 Zabrze, Poland; 3 Student Scientific Society, University Department of Cardiac Anaesthesia and Intensive Care, Faculty of Medical Sciences in Zabrze, Medical University of Silesia, Plac Traugutta 2 St., 41-800 Zabrze, Poland; 4 Department of Anaesthesiology and Intensive Therapy, Faculty of Medical Sciences in Zabrze, Medical University of Silesia, Plac Traugutta 2 St., 41-800 Zabrze, Poland; 5 Department of Cardiac Anaesthesia and Intensive Care, Silesian Center for Heart Diseases in Zabrze, Faculty of Medical Sciences in Zabrze, Medical University of Silesia, Plac Traugutta 2 St., 41-800 Zabrze, Poland; 6 Department of Epidemiology, Faculty of Medical Sciences in Katowice, Medical University of Silesia, Medyków 14 St., 40-752 Katowice, Poland; 7 Department of Lung Diseases and Tuberculosis, Faculty of Medical Sciences in Zabrze, Medical University of Silesia, Plac Traugutta 2 St., 41-800 Zabrze, Polandsimon.mds@poczta.fm (S.S.); 8 Department of Anesthesiology and Intensive Therapy, Healthcare Center of the Ministry of Internal Affairs and Administration Named After Sergeant Grzegorz Załoga, Wita Stwosza 39-41 St., 40-042 Katowice, Poland

**Keywords:** therapeutic plasma exchange (TPE), intensive care units (ICU), complications, APACHE, SOFA

## Abstract

(1). Background: Therapeutic plasma exchange (TPE) is a method of extracorporeal plasma filtration designed to remove immunoglobulins and pro-inflammatory factors as pathogenesis of numerous diseases. Procedures are performed in intensive care units (ICUs); however, the complications and treatment outcomes remain unclear. The aim of the study was to evaluate clinical outcomes and identify risk factors of complications associated with TPE. (2). Methods: In this multi-center, retrospective, 5-year cohort study, we analyzed a database of 56 adult ICU patients who had undergone TPE at two academic hospitals and one regional hospital. (3). Results: In our study, the median APACHE II was 7.5 IQR 12.5 (4–16.5) and SOFA score was 2 IQR 4 (1–5). In the multivariate analysis, the APACHE II (*p* = 0.043) and SOFA score (*p* = 0.046) were the predictors of prolonged length of stay. The SOFA score was a predictor of hospital-acquired infection (HAI) (*p* = 0.011). Multivariate logistic regression revealed age (*p* = 0.011), SOFA (*p* = 0.011), and APACHE II score (*p* = 0.032) as independent predictors of the development of shock as a TPE complication. (4). Conclusions: Our results suggest that the SOFA and APACHE II scores are significant predictors of the length of hospitalization and the occurrence of shock. In addition, the SOFA score was a predictor of HAI in patients treated TPE in ICU.

## 1. Introduction

Therapeutic plasma exchange (TPE), also known as therapeutic plasmapheresis (TP), is a method of extracorporeal plasma filtration designed to remove immunoglobulins (mainly autoantibodies) and pro-inflammatory factors (cryoglobulins, lipoproteins, immune complexes, immunoglobulin light chains), which are crucial for the pathogenesis of numerous diseases such as myasthenia gravis (MG) or Guillain–Barré syndrome (GBS) [1]. The indications for plasmapheresis are based on recommendations of the working groups consisting of specialists in various fields using extracorporeal blood purification techniques. The most recent American Society for Apheresis (ASFA) guidelines (2023) highlighted 77 diseases with 119 indications for TPE, including 20 indications with TPE listed as a first-line treatment (ASFA category I) and 23 indications as a second-line treatment (ASFA category II) [2,3].

The procedure consists of separating plasma and hence eliminating the substances of high molecular weight, returning the blood morphotic elements and supplementing the fluid volumes lost, and it has two mechanisms of action: removal of a pathogenic substance from the plasma and delivery of deficient plasma components [4]. Generally, the whole blood from the peripheral or central patient’s vascular access is pumped inside the plasma separator [5] and filtered by centrifugal separation (cTPE) or membrane separation (mTPE) [6]. After both cTPE and mTPE separation, the liquid fraction of the blood and smaller molecules are separated, forming part of the waste plasma. cTPE or mTPE involves separating patient’s plasma from other components of blood to remove pathological factors like autoimmune antibodies, donor-specific antibodies, cytokines, excessive paraproteins and endogenous and exogenous toxins (e.g., IgG in MG, anti-GM1, anti-GD1a antibodies in GBS, IgM in Waldenström macroglobulinemia, or IgG and IgM iso-agglutinins prior to ABO incompatible organ transplantation, anti-aquaporin IgG in neuromyelitis optica) [7,8,9].

TPE also gives the possibility to replenish deficient factors like clotting factors or replacing plasma components as in thrombotic thrombocytopenic purpura (TTP) (ADAMTS13) [4,6]. Plasma can be completely eliminated and replaced in equal volume with a replacement solution, or plasma can be further processed by removing pathogens with specific binding agents [5].

The remaining cell-rich blood is mixed with the sterile, nonpyrogenic, isovolemic, allergen-free, isosmotic, replacement fluid to maintain hemodynamic stability and prevent hypovolemia [4,5]. The fluid replacements are crystalloid (physiological or electrolytic solution) and colloids solution (mainly albumin or fresh frozen plasma [FFP]) [5]. Equally important in TPE is anticoagulation, as it provides a balance in preventing bleeding and loss of vital blood components by ensuring uninterrupted flow in the extracorporeal circuit, minimizing unintended loss of valuable blood components due to procedure-related interruptions. Heparin and citrate are the two most common anticoagulants used [4,10,11].

Therapeutic plasma exchange is also a vital therapeutic procedure in the intensive care unit (ICU) for managing multiple disorders, some of which include the hematologic, autoimmune, and neurological systems [6,12]. Plasma exchange therapy has become a point of interest and, over time, the standard of care for various diseases since the introduction of automated cell separators in the 1970s. Studies have shown the safety of its use with a 4.75% overall incidence of mostly reversible side effects [13,14]. However, patients in the ICU are often hemodynamically unstable due to their underlying conditions, and plasmapheresis can further contribute to hypotension and electrolyte imbalances, particularly hypocalcemia and hypokalemia, leading to cardiac arrhythmias. Another potential risk is allergic and anaphylactic reactions due to exposure to donor plasma and red blood cells. ICU patients are also prone to coagulopathies, and those undergoing plasmapheresis may experience decreased levels of hemoglobin, platelets, and fibrinogen, which can further aggravate coagulopathies [6]. The challenges include possible worsening of the hemodynamics, electrolyte imbalances—such as calcium, magnesium, zinc, and selenium—the unintended removal of proteins that transport micronutrients (e.g., ceruloplasmin, transferrin), coagulopathies, and infection risks.

To assess the relevance of risk factors and complications of TPE in ICU, we conducted a five-year retrospective multicenter study. The primary endpoint was to assess clinical outcome, while the secondary endpoint was to circle the type of possible complications associated with the procedure.

## 2. Materials and Methods

### 2.1. Study Design

This retrospective analysis included 56 patients who had undergone the TPE procedure in ICUs at two academic hospitals—Silesian Center for Heart Diseases in Zabrze, Poland, and Independent Public Clinical Hospital No. 1 named after Prof. Stanislaw Szyszko—and one regional ministry hospital—Healthcare Center of the Ministry of Internal Affairs and Administration named after Sergeant Grzegorz Załoga in Katowice, Poland—between January 2019 and November 2024. In all centers participating in the study, TPE therapies were performed based on ASFA guidelines, including indications for treatment, volume of plasma removed, frequency, and number of procedures performed.

The amount of plasma removed was calculated based on the patient’s calculated plasma volume (PV). PV was calculated using the most commonly used formula: PV = body weight (kg) × 0.065 × (1-hematocrit) [5]. Typically, 1–1.5 PV was removed per plasmapheresis session. Plasmapheresis was repeated several times, usually 4–5 TP every 24–48 h.

### 2.2. The Inclusion Criteria

The inclusion and exclusion criteria in our study covered all adult (>18 years of age) patients admitted to the ICU for TPE, or patients who required this procedure during their hospitalization. Pediatric patients were excluded from the study due to the nature of the centers where TPE was performed.

The study included all TPE sessions (2 to 6 sessions) during the patient’s hospitalization. In terms of the functioning of our departments in our centers, none of our patients were rehospitalized during the period covered by our study.

### 2.3. Statistical Analysis

Statistical analyses were performed using the procedures of the Statistica^®^ software, version 13.3 (StatSoft, Kraków, Poland). The distribution of quantitative variables was presented as the mean values and their standard deviations or median and quartile range in case of the categorical values were presented as absolute (n) and relative frequencies (%). Normality of distributions of continuous variables was assessed by the Shapiro–Wilk test. Statistical significance of differences between continuous variables was analyzed by the independent samples *t*-test or analysis of variance (ANOVA). If the assumptions for these were not met, the Mann–Whitney U test or Kruskal–Wallis test was used. Between-group differences in the distribution of qualitative variables were tested by the chi-square test. Depending on normality distribution, correlations were based on the results of Pearson’s or Spearman’s correlation.

In the multivariate analysis, logistic regression models were constructed to identify independent predictors of significant treatment outcomes. Variables that showed statistical significance in the univariate analysis (*p* < 0.05) were included in the initial multivariate model. Additionally, covariates known from prior literature to be established predictors of treatment response were also incorporated, regardless of their univariate significance, to control for potential confounding effects. Model selection was guided by clinical relevance, statistical criteria, and considerations of multicollinearity.

According to our regulations, such retrospective analysis did not require Bioethics Committee approval (BNW/NWN/0052/KB/231/25).

## 3. Results

### 3.1. General Data

In our analyzed group, there were 56 patients (17 women, 39 men) undergoing the TPE procedure. The mean age of patients was 54.7 (18–81).

In total, 46 (82.14%) patients were conscious on admission to ICU. Patients were characterized by cardiac insufficiency (6, 10.71%) and respiratory failure (17, 30.36%) (Table 1). Median length of hospitalization was 7 days IQR 11.5 (6.5–18).

All patients were assessed with the use of Acute Physiology and Chronic Health Evaluation II (APACHE II), Sequential Organ Failure Assessment (SOFA) and Charlson Comorbidity Index (CCI) scales (Table 2).

### 3.2. TPE

Patients underwent TPE due to multiple indications (shown in Table 3). In addition to the main indications for TPE, respiratory failure was also an additional indication in 15 (26.79%) patients.

The TPE procedure in patients was performed in most cases: five times (IQR = 1 (3–5)) in 32 (57.14%) patients (Table 4).

To determine the volume of plasma to be exchanged during TPE, the following formula was used to estimate the effective plasma volume (EPV): EPV = [0.065 × body weight (kg)] × [1 − hematocrit (Hct)], where 0.065 represents an approximate value for total blood volume per kilogram of body weight (i.e., ~65 mL/kg), and [1 − Hct] reflects the plasma fraction of total blood volume (i.e., the non-cellular component) [15]. It allows for the exchange of approximately 40–50 mL of plasma per kilogram of body weight during a single procedure, corresponding to roughly 2.800 to 3.500 mL for a patient. The average amount of plasma transfused during TPE was 15 l ± 4.2 (6–22.3). All TPEs were performed under ICU isolation to minimize the risk of infection for patients undergoing TPE.

### 3.3. Complications

TPE complications occurred in 33 (58.92%) patients. The most common complication was hypotension, which was observed in 29 (51.78%) patients. Other complications included exacerbation of circulatory failure, cardiac arrhythmias (bradycardia, tachycardia), tachypnea, desaturation during procedure, abdominal pain, nausea and vomiting, muscle tremors, increased sweating, weakness and hematoma at the vascular access site (Table 5).

### 3.4. Pre-Hospital Infection

Pre-hospital infection (<48 h before admission) was present in 22 (39.28%) patients, and hospital-acquired infections (HAIs) were present in 19 (33.92%) of the group. Additionally, 12 (21.42%) patients developed infectious complications after undergoing TPE.

In total, 39 (69.64%) patients received antibiotic therapy in the ICU. Patients who did not require treatment with metronidazole, vancomycin, or piperacillin-tazobactam clinically improved more significantly following TPE compared to those who required treatment with these antibiotics (*p* < 0.01).

### 3.5. Oxygen Therapy

In our study, 40 patients required oxygen therapy, while 16 patients required mechanical ventilation (respiratory therapy).

Patients qualified for TPE due to severe manifestations of MG required oxygen therapy in the ICUs more than seven times more frequently than those treated for other indications (*p* < 0.05), and mechanical ventilation was necessary 16 times more often compared to patients with GBS and neuromyelitis optica, respectively (*p* = 0.0007).

It is noteworthy that, in comparison to the other two conditions, exacerbations in the course of MG may present with significantly greater severity, often necessitating advanced medical interventions in the ICU setting. However, this patient population may also derive substantial benefit from TPE, which can contribute to therapeutic de-escalation and clinical stabilization.

### 3.6. Condition After TPE—Improvement/Mortality

Clinical improvement after TPE was observed in 42 (75%) patients. Clinical improvement is defined as stabilization of the patient’s condition or objective and subjective improvement in the patient’s overall condition, including improvement in respiratory and circulatory function, neurological status (including mobility, muscle strength, speech and swallowing) and vision.

In a group of patients with MG, nearly 9 times more improvements in the general condition of patients were observed compared to other indications for TPE (OR) of 8.9 (95% CI 1.07–74.9) (*p* < 0.05).

Mortality occurred in 5 (8.92%) patients. One (2.43%) patient died in the course of progressive underlying disease with no observed improvement after TPE (complication of shock). The other four (7.14%) patients had respiratory failure on admission with underlying diseases such as COVID-19 in two patients, MG and GPA.

### 3.7. Multivariate Analysis

The multivariate analysis included the following as the independent variables: age, sex (male versus female), APACHE II score, SOFA score, CCI, co-existing diseases as heart failure, myocardial infarction, arterial hypertension, type 2 diabetes, and chronic kidney disease.

In the multivariate analysis, the APACHE II score [*p* = 0.043, 95% CI (−0.99)–(−0.01)] and SOFA score [*p* = 0.046, 95% CI (−1.81)–(−0.01)] were the sole predictors of the length of hospitalization. An increase in either the APACHE II or the SOFA score was associated with a prolonged hospital stay.

In the multivariate analysis, the SOFA score was identified as a predictor of hospital-acquired infection [(*p* = 0.011, 95% CI (−1.07)–(−0.13)]. Higher SOFA scores were associated with an increased risk of hospital-acquired infections.

Multivariate logistic regression revealed age [*p* = 0.011, 95% CI (−0.25–(−0.32)], SOFA score [(*p* = 0.011, 95% CI (−0.25)–(−0.03)], and APACHE II score (*p* = 0.032, 95% CI 0.03–0.67) as independent predictors of the development of shock as a complication following TPE.

Higher values of these parameters were significantly associated with an increased incidence of shock and a consequent need for vasopressor support.

Multivariate analysis did not identify any predictors of mortality.

## 4. Discussion

One of the most important findings from our study, involving patients undergoing TPE in the ICUs, is that both the SOFA and APACHE II scores serve as significant predictors of critical in-hospital outcomes, including the length of hospitalization and the occurrence of shock, while the SOFA score alone was identified as a predictor of hospital-acquired infections. Additionally, advanced age was independently associated with an increased risk of shock.

Higher values of these clinical scores were consistently linked to prolonged hospital stays, greater susceptibility to nosocomial infections (in the case of the SOFA score), and an increased need for vasopressor support, underscoring their prognostic value in the management of ICU patients undergoing plasmapheresis.

### 4.1. TPE in ICU

Surprisingly most patients requiring TPE are not in critical condition and do not need to be admitted to the ICU and can be admitted to another internal medicine department (hematology, nephrology) to perform this technique. Therefore, a study in Madrid shows the wide availability of performing TPE in the ICU in terms of time (53.8% of ICUs available 24 h a day, 365 days a year) in comparison to other internal medicine departments, where TPE is typically performed on a scheduled basis [16]. The ICU offers more availability, allowing the procedure to be carried out urgently when needed, the availability of trained specialists and performing TPE not only in critical cases, but in any patient [16]. Moreover, there is no need to move the patient, and timely and optimal treatment can be provided every time [16]. In contrast, patients in our study required transfer to the intensive care unit for the TPE procedure due to deterioration of their condition.

Patients typically admitted to ICUs differ significantly from those hospitalized solely for the purpose of undergoing plasmapheresis. Our findings show that the median ICU stay in our cohort was 7 days, compared to the worldwide average of 11 days mentioned in a large study in 57 countries [17]. Importantly, the length of stay in the ICU is directly associated with an increased risk of nosocomial infections, which—when leading to sepsis—can substantially elevate ICU mortality rates [18,19].

Notably, the severity scores most commonly used in the ICU setting—such as SOFA and APACHE II—indicate a significantly different risk profile in patients admitted for plasmapheresis compared to the general ICU population. In a large cohort study involving over 100,000 ICU patients, the mean SOFA score was 7.5 ± 3.6 for women and 7.8 ± 3.6 for men [20]. This contrasts sharply with the mean SOFA score of 3.42 and a median of 2 (IQR 4; 1–5) observed in our plasmapheresis group. This discrepancy may be attributed to the organizational structure of the centers included in our study, where TPE procedures are not performed in internal medicine wards. As a result, patients referred for plasmapheresis to the ICU may not present with the same level of critical illness as the broader ICU population, and their relatively low SOFA scores suggest that, from a clinical standpoint, they could potentially be managed in a non-ICU setting under different institutional circumstances.

Similarly, the APACHE II score was significantly higher in typical Polish ICU patients—median of 9 (IQR 12; 12–24)—compared to patients admitted for plasmapheresis, who had a median of 7.5 (IQR 12.5; 4–16.5) [20].

ICUs are hospital departments where approximately 30% of all nosocomial infections occur [21], placing patients at significant risk of complications, including sepsis. As a result, broad-spectrum antibiotic therapy is frequently initiated. In our cohort, nearly 70% of patients received antibiotic treatment, although only 55% presented with signs of infection prior to admission. This suggests that approximately 15% of patients developed infections during their ICU stay, which might have been avoidable had the procedure been performed in a unit with a lower incidence of HAIs.

These findings indicate that, in patients being considered for ICU admission solely for TPE, the procedure could potentially be performed in an appropriately equipped non-ICU medical ward, where the clinical environment may offer a lower risk of complications. This approach warrants further evaluation in future studies. Given the relatively stable condition of most patients and their lower severity scores, performing the procedure in a nephrology unit would likely reduce the risk of nosocomial infections, which remain a common and serious complication in ICU settings [22].

### 4.2. SARS-CoV-2 Infection Indication (COVID-19)

With the advent of the pandemic in 2020, the lack of effective treatments has led to the development of new therapeutic strategies and consideration of the wisdom of performing TPE in patients with COVID-19 [23,24]. Nowadays, there are several studies [25,26,27,28] which indicate the effectiveness and benefit of using plasmapheresis for prevention and treatment of patients with COVID-19. In our study, in two (3.57%) patients, this disease was the head cause for performing TPE. Unfortunately, despite the use of TPE, both patients with COVID-19 with associated respiratory failure died after the second cycle of TPE, and their hospitalization times were 30 and 32 days. In contrast, Fonseca-González et al. report that TPE reduced the 60-day mortality rate for COVID-19 compared with the use of standard therapy (20% vs. 50%) [29]. In the randomized controlled trial, Faqihi et al. report reduced 35-day mortality in patients with life-threatening COVID-19 (20.9% vs. 34.1%) [30]. One meta-analysis confirmed the validity of considering TPE use in patients with moderate-to-critical COVID-19 due to significantly reduced mortality (17.92% vs. 44.71%) [31]. Such substantial differences may be attributed to the relatively small cohort of patients with COVID-19 included in our study. Notably, these two patients were in a more severe clinical condition due to their overall systemic status, which may explain the absence of a reduction in mortality rate for COVID-19 in our cohort compared to the findings reported in the aforementioned studies.

### 4.3. Differences in Guidelines, Types of Pump, Equipment, Differences—What Effects with Different Uses?

In our study, approximately 3 L of plasma was transfused in each procedure, which is in accordance with the literature, in order to remove an average of 65–70% of target substances from the intravascular compartment [32].

Cervantes et al. emphasize that effective TPE should consist of four to five consecutive sessions administered every 24–48 h to achieve meaningful clearance, potentially removing up to 90% of pathogenic substances [12,32].

In our study, the majority of patients underwent TPE in accordance with recommendations, with five sessions administered in 32 patients (57.14%) [IQR = 1; range 3–5]. Additionally, 4 patients (7.1%) received six sessions, 10 patients (17.86%) received four, 7 patients (12.5%) underwent three sessions, and 3 patients (5.35%) received two.

### 4.4. Complications in TPE

Complications during TPE are relatively rare at 4–5%, of which 0.025–0.2% are life-threatening ones [3,33]. Kiprov et al. analyzed a total of 17,940 TPE procedures performed in 3583 patients, reporting adverse reactions in 3.9% of all sessions [34].

Bauer et al. report that the complications related to the procedure itself—most commonly associated with vascular access, the choice of replacement fluid, or the anticoagulation method—occur in approximately 5% to 36% of cases, making them the most frequent adverse events related to TPE [4].

Courier et al. indicated that approximately 91% of complications were classified as mild (did not require medical intervention or delay the procedure) or moderate–severe (required medical intervention and/or delayed a procedure) [35].

Basic-Jukin et al. analyzed data from 509 patients who underwent a total of 4857 plasma exchange procedures, reporting 231 adverse reactions—corresponding to an overall complication rate of 4.75% per treatment session. The most commonly observed complications included paresthesias (2.7%), hematoma at the puncture site (2.4%), circuit clotting (1.7%), mild to moderate allergic reactions such as urticaria (1.6%), and bleeding (0.06%) [33].

In our study, TPE complications occurred in 33 (58.92%) patients, and the most common complication was hypotension, which was observed in 29 (51.78%) patients.

Mild complications include paresthesia [35] and muscle cramps caused by a decrease in calcium ion concentration—which is the most common complication after TPE [36,37]. Additionally, they include urticaria, dizziness, nausea, vomiting, hypotension (which was the most common in our study), hypofibrinogenemia and metabolic ABN: hypocalcemia, hypophosphatemia and hypomagnesemia [3,35]. Bauer et al. indicated that risk factors of hypocalcemia are citrate anticoagulation and plasma replacement [3,4].

In our study, in three (5.36%) patients, calcium imbalance was observed. In addition, we noted cardiac arrhythmias such as bradycardia in 10 (17.8%) and tachycardia in 7 (12.5%). Moreover, coagulation disorders were observed in three (5.36%) patients.

Symptoms similar to anaphylaxis, such as flushing, low blood pressure, abdominal cramping, and other gastrointestinal issues, have infrequently been reported in patients who took an angiotensin-converting enzyme (ACE) inhibitor within 24 to 30 h prior to undergoing plasmapheresis and were given albumin for fluid replacement [38].

However, life-threatening complications are often associated with the critically severe condition of the patient before the procedure and are dominated by anaphylactoid reactions and severe hypotension [3,4].

Kiprov et al. observed that the most common complications associated with therapeutic plasma exchange included reactions related to acid citrate dextrose (ACD) toxicity (3%), vasovagal episodes (0.5%), and also adverse reactions to FFP (0.12%) [34].

Basic-Jukin et al. reported that, in their cohort, true anaphylactoid reactions occurred in five procedures, and the incidence of severe, potentially life-threatening adverse events was 0.12% per treatment session [33].

In our study, one patient decompensated and collapsed during the procedure, so life-threatening reversal management was implemented. Moreover, there are (hemo)pneumothorax, catheter-related bacteremia and hamartoma [35].

### 4.5. APACHE II, SOFA and CCI Scales

As predictive factors and occurrence complications in patient groups treated with plasmapheresis in ICU.

In our cohort of patients treated with TPE, significant correlations were observed between higher APACHE II, SOFA, and CCI scores and the occurrence of HAIs (*p* = 0.0009, *p* = 0.0002, and *p* = 0.028, respectively). Notably, the APACHE II and SOFA scores showed a specific association with severe infections, including pneumonia, urinary tract infections, and sepsis following plasmapheresis (*p* = 0.039 and *p* = 0.022, respectively).

Additionally, in our study, the SOFA score was identified as an independent predictor of HAIs in the ICU (*p* = 0.011). Higher SOFA scores were associated with an increased risk of these infections, highlighting that plasmapheresis performed in the ICU setting—where the incidence of HAIs is difficult to avoid—can lead to potentially life-threatening complications.

In the Polish healthcare setting, the most prevalent causes of HAIs include Klebsiella pneumoniae, Escherichia coli, Acinetobacter baumannii, and Clostridium difficile [39]. Our analysis showed that higher scores on the APACHE II and SOFA scales were significantly correlated with infections caused by K. pneumoniae, E. coli and A. baumannii (considered multidrug-resistant pathogens), suggesting that severity of illness, as reflected by these scoring systems, may be associated with increased susceptibility to such infections.

Moreover, our results suggest the usefulness of these three scales in predicting the need for active respiratory support for patients undergoing TPE. Patients with higher APACHE II, SOFA, and CCI scores were more likely to require oxygen supplementation in the ICU setting (APACHE II: *p* < 0.01; SOFA: *p* < 0.01; CCI: *p* < 0.01). A similar trend was noted for the initiation of invasive mechanical ventilation (APACHE II: *p* < 0.01; SOFA: *p* < 0.01; CCI: *p* < 0.01).

In addition, in our patients, a statistically significant association was observed between SOFA score and the development of shock as a complication following TPE, with vasopressor support (*p* < 0.05), highlighting the need for close clinical monitoring during therapy and the use of appropriate treatment.

These findings highlight that the APACHE II and SOFA scales may serve as valuable tools for assessing key risks associated with TPE in the ICU, including infections, shock (in the case of the SOFA score) and extended hospitalization.

Current evidence suggests that the initial cycles of TPE in critically ill patients should be performed in the ICU, whereas routine cycles—for example, in chronic neurological or nephrological conditions—can be safely conducted in specialized wards such as nephrology or neurology [5,40,41]. This approach may reduce ICU-associated risks, including nosocomial infections. Pham et al. classify TPE patients as emergent, urgent, or routine, supporting ICU care for severe cases and non-ICU settings for routine procedures [41]. However, larger multicenter studies are needed to further evaluate the safety and efficacy of TPE outside the ICU.

Regarding our study, a novel approach could be considered, whereby patients with low SOFA and APACHE II scores (referred to as low-risk patients) and patients with routine cycles of TPE could undergo TPE in nephrology or internal medicine wards instead of ICU. However, as multivariate analysis did not identify any predictors of mortality, these scoring systems—though helpful in evaluating patients’ clinical status—should be complemented by close clinical monitoring and individualized patient assessment throughout the course of treatment.

### 4.6. Limitations

The main limitations of our study include the fact that this is a retrospective study, so there is a possibility of missing data, which may be important in small quantitatively sparse populations such as the one analyzed, and low power of analysis. Furthermore, all three centers performed TPE in the ICU, but differed in profile, type of equipment, and technical and staffing capacity (replacement fluid, anticoagulation methods, and catheter-related complications). Additionally, heterogeneity in the indications for TPE is evident; however, the majority of patients included in the study had neurological diagnoses. A potential limitation is that part of the period analyzed was a pandemic, which may have affected the patient profile and resulted in the treatment of patients in the acute phase of COVID-19.

## 5. Conclusions

TPE is a standard, life-saving intervention in ICUs for treating neurological, nephrological, and pulmonary complications of autoimmune diseases. It is generally safe, with a low complication rate, most commonly involving cardiovascular, respiratory, and electrolyte disturbances. The ICU setting provides optimal conditions for safe plasmapheresis, with continuous monitoring, immediate intervention, and access to life-support measures, equipment, and trained medical staff. However, it also poses a risk of hospital-acquired infection caused by multidrug-resistant strains of bacteria. Comparing outcomes across departments could help optimize safety further based on infrastructure and available resources. Our study provides additional insight, suggesting the possibility of identifying significant predictive markers for complications associated with plasmapheresis therapy in critically ill patients.

## Figures and Tables

**Table 1:** Study group characteristics. Descriptive and demographic characteristics of patients.

Study Group Characteristics	
Patient Characteristics	Results
Age (years)	54.7 (18–81)
Sex	17 women (30.35%), 39 men (69.64%)
Patient conscious (on admission) n (%)	46 (82.14%)
Cardiac failure (on admission) n (%)	6 (10.71%)
Respiratory failure (on admission) n (%)	17 (30.36%)
Complications after TPE n (%)	33 (58.92%)
Antibiotic therapy n (%)	39 (69.64%)
Clinical improvement after TPE n (%)	42 (75%)

**Table 2:** Scales. Characteristics of patients.

Scales	
Scale	Results
CCI	2 IQR 3 (1–4)
CCI%	90 IQR 43 (53–96)
APACHE II	7.5 IQR 12.5 (4–16.5)
SOFA	2 IQR 4 (1–5)

**Table 3:** Indications for therapeutic plasma exchange (TPE).

Indications for Therapeutic Plasma Exchange (TPE)	
Type of Indication (Diagnosis)	Number of Patients
Myasthenia gravis (MG)	17 (30.36%)
Guillain–Barré syndrome (GBS)	16 (28.57%)
Neuromyelitis optica (NMO)	7 (12.5%)
Granulomatosis with polyangiitis (GPA)	5 (8.92%)
Another type of indications (diagnosis)	
SARS-CoV-2 co-infection	2 (3.57%)
Transverse myelitis (TM)	2 (3.57%)
Acute Disseminated Encephalomyelitis (ADEM)	1 (1.79%)
Autoimmune hemolytic anemia with the presence of IgG3 antibodies (AIHA)	1 (1.79%)
Tick-borne encephalitis virus (TBEV)	1 (1.79%)
Multiple sclerosis (MS)	1 (1.79%)
Hemolytic uremic syndrome (HUS) and Thrombotic thrombocytopenic purpura (TTP)	1 (1.79%)
Post-transplant HLA antibodies with a positive cross-match	1 (1.79%)
Polyneuropathy of uncertain etiology (PN)	1 (1.79%)
Respiratory failure (RF)	15 (26.79%)

**Table 4:** TPE procedure characteristics.

TPE Procedure Characteristics	
Number of Cycles in TPE Procedure	Number of Patients
6	4 (7.1%)
5	32 (57.14%)
4	10 (17.86%)
3	7 (12.5%)
2	3 (5.35%)

**Table 5:** Complications after TPE.

Complications After TPE	
Type of Complication	Number of Patients
Hypotension	29 (51.78%)
Bradycardia	10 (17.8%)
Tachycardia	7 (12.5%)
Hematoma	3 (5.36%)
Coagulation disorders	3 (5.36%)
Calcium imbalance	3 (5.36%)

## Data Availability

The medical records from which the data were obtained, as well as the components of the research database, are located within the IT systems of each of the hospital centers included in the retrospective analysis. A significant portion of the data also exists solely in paper format. Therefore, the research database was not transmitted to any cloud-based platform and is not available online. Further inquiries can be directed to the corresponding author.

## Abbreviations

The following abbreviations are used in this manuscript:

TPE	Therapeutic plasma exchange
TP	Therapeutic plasmapheresis
MG	Myasthenia gravis
GBS	Guillain–Barré syndrome
ASFA	American Society for Apheresis
cTPE	Centrifugal separation TPE
mTPE	Membrane separation TPE
TTP	Thrombotic thrombocytopenic purpura
FFP	Fresh frozen plasma
ICU	Intensive care unit
APACHE II	Acute Physiology and Chronic Health Evaluation II
SOFA	Sequential Organ Failure Assessment
CCI	Charlson Comorbidity Index
NMO	Neuromyelitis optica
GPA	Granulomatosis with polyangiitis
ADEM	Acute Disseminated Encephalomyelitis
AIHA	Autoimmune hemolytic anemia with the presence of IgG3 antibodies
TM	Transverse myelitis
TBEV	Tick-borne encephalitis virus
MS	Multiple sclerosis
HUS	Hemolytic uremic syndrome
RF	Respiratory failure
PN	Polyneuropathy of uncertain etiology
EPV	Effective plasma volume
Hct	Hematocrit
HAIs	Hospital-acquired infections
DAH	Diffuse alveolar hemorrhage
NMOSD	Neuromyelitis optica spectrum disorders
ON	Optic neuritis
RPGN	Rapidly progressive glomerulonephritis
CP	Convalescent plasma
ACE	Angiotensin-converting enzyme
ACD	Acid citrate dextrose

## References

[1] Szczeklik W., Mitka I., Nowak I., Seczyńska B., Sega A., Węgrzyn W., Królikowski W. (2010). Plazmafereza w oddziale intensywnej terapii [Plasmapheresis in intensive therapy unit]. Anestezjol. Intens. Ter..

[2] Connelly-Smith L., Alquist C.R., Aqui N.A., Hofmann J.C., Klingel R., Onwuemene O.A., Patriquin C.J., Pham H.P., Sanchez A.P., Schneiderman J. (2023). Guidelines on the Use of Therapeutic Apheresis in Clinical Practice—Evidence-Based Approach from the Writing Committee of the American Society for Apheresis: The Ninth Special Issue. J. Clin. Apher..

[3] Wiśniewska B. (2018). Therapeutic plasmapheresis in clinical practice. Hematologia.

[4] Bauer P.R., Ostermann M., Russell L., Robba C., David S., Ferreyro B.L., Cid J., Castro P., Juffermans N.P., Montini L. (2022). Plasma exchange in the intensive care unit: A narrative review. Intensive Care Med..

[5] Altobelli C., Anastasio P., Cerrone A., Signoriello E., Lus G., Pluvio C., Perna A.F., Capasso G., Simeoni M., Capolongo G. (2023). Therapeutic Plasmapheresis: A Revision of Literature. Kidney Blood Press. Res..

[6] Hussein G., Liu B., Yadav S.K., Warsame M., Jamil R., Surani S.R., Khan S.A. (2023). Plasmapheresis in the ICU. Medicina.

[7] David S., Russell L., Castro P., an de Louw A., Zafrani L., Pirani T., Nielsen N.D., Mariotte E., Ferreyro B.L., Kielstein J.T. (2023). Research priorities for therapeutic plasma exchange in critically ill patients. Intensive Care Med. Exp..

[8] Naciri Bennani H., Noble J., Chevallier E., Terrec F., Motte L., Giroux-Lathuile C., Bugnazet M., Imerzoukene F., Janbon B., Malvezzi P. (2021). Isoagglutinin removal by plasma centrifugation or filtration (single or double): A prospective study in a single center. J. Clin. Apher..

[9] Siemiński M. (2012). Immunoglobuliny w terapii zespołu Guillaina-Barrégo. Pol. Przegląd Neurol..

[10] Stegmayr B., Ptak J., Wikström B., Berlin G., Axelsson C.G., Griskevicius A., Centoni P., Liumbruno G., Molfettini P., Audzijoniene J. (2008). World apheresis registry 2003–2007 data. Transfus. Apher. Sci..

[11] Kissling S., Legallais C., Pruijm M., Teta D., Vogt B., Burnier M., Rondeau E., Ridel C. (2017). A new prescription model for regional citrate anticoagulation in therapeutic plasma exchanges. BMC Nephrol..

[12] Domínguez A., Fraga C.R., Ayala R., Conde P., García D., Cereijo A. (2025). Cerebral vasculitis secondary to pneumococcal meningitis. Plasmapheresis as adjuvant therapy. Case report. Anaesthesiol. Intensive Ther..

[13] Brecher M.E. (2002). Plasma exchange: Why we do what we do. J. Clin. Apher..

[14] McLeod B.C., Sniecinski I., Ciavarella D., Owen H., Price T.H., Randels M.J., Smith J.W. (1999). Frequency of immediate adverse effects associated with therapeutic apheresis. Transfusion.

[15] Kaplan A.A. (2013). Therapeutic plasma exchange: A technical and operational review. J. Clin. Apher..

[16] Alonso-Ovies Á., Álvarez Rodríguez J. (2023). Plasmapheresis in the intensive care units of the community of Madrid. Med. Intensiv..

[17] Khanna A.K., Labeau S.O., McCartney K., Blot S.I., Deschepper M., DecubICUs study Team, the European Society of Intensive Care Medicine (ESICM) Trials Group collaborators (2022). International variation in length of stay in intensive care units and the impact of patient-to-nurse ratios. Intensive Crit. Care Nurs..

[18] Jeon C.Y., Neidell M., Jia H., Sinisi M., Larson E. (2012). On the role of length of stay in healthcare-associated bloodstream infection. Infect. Control. Hosp. Epidemiol..

[19] Markwart R., Saito H., Harder T., Tomczyk S., Cassini A., Fleischmann-Struzek C., Reichert F., Eckmanns T., Allegranzi B. (2020). Epidemiology and burden of sepsis acquired in hospitals and intensive care units: A systematic review and meta-analysis. Intensive Care Med..

[20] Godinjak A., Iglica A., Rama A., Tančica I., Jusufović S., Ajanović A., Kukuljac A. (2016). Predictive value of SAPS II and APACHE II scoring systems for patient outcome in a medical intensive care unit. Mortality.

[21] Magill S.S., Edwards J.R., Bamberg W., Beldavs Z.G., Dumyati G., Kainer M.A., Lynfield R., Maloney M., McAllister-Hollod L., Nadle J. (2014). Multistate point-prevalence survey of health care-associated infections. N. Engl. J. Med..

[22] Blot S., Ruppé E., Harbarth S., Asehnoune K., Poulakou G., Luyt C.E., Rello J., Klompas M., Depuydt P., Eckmann C. (2022). Healthcare-associated infections in adult intensive care unit patients: Changes in epidemiology, diagnosis, prevention and contributions of new technologies. Intensive Crit. Care Nurs..

[23] Cagdas D. (2021). Convalescent plasma and hyperimmune globulin therapy in COVID-19. Expert Rev. Clin. Immunol..

[24] Yiğenoğlu T.N., Hacıbekiroğlu T., Berber İ., Dal M.S., Baştürk A., Namdaroğlu S., Korkmaz S., Ulas T., Dal T., Erkurt M.A. (2020). Convalescent plasma therapy in patients with COVID-19. J. Clin. Apher..

[25] Pourahmad R., Moazzami B., Rezaei N. (2020). Efficacy of Plasmapheresis and Immunoglobulin Replacement Therapy (IVIG) on Patients with COVID-19. SN Compr. Clin. Med..

[26] Dianaty S., Khodadadi S., Alimoghaddam R., Mirzaei A. (2023). Comparison of outcomes and costs of extracorporeal blood purification therapies in critically ill COVID-19 patients. Ther. Apher. Dial..

[27] Rafieian S., Saghafi F., Birjandi B., Rafieian S., Dehghanpour H., Khalaj F., Zare S., Chenari H.D., Hajimaghsoudi M., Sohrevardi S.M. (2023). Efficacy and safety of Tocilizumab, plasmapheresis and their combination in severe COVID-19: A randomized clinical trial. Int. Immunopharmacol..

[28] Balagholi S., Dabbaghi R., Eshghi P., Mousavi S.A., Heshmati F., Mohammadi S. (2020). Potential of therapeutic plasmapheresis in treatment of COVID-19 patients: Immunopathogenesis and coagulopathy. Transfus. Apher. Sci..

[29] Fonseca-González G., Alamilla-Sánchez M., García-Macas V., Herrera-Acevedo J., Villalobos-Brito M., Tapia-Rangel E., Maldonado-Tapia D., López-Mendoza M., Cano-Cervantes J.H., Orozco-Vázquez J. (2023). Impact of plasmapheresis on severe COVID-19. Sci. Rep..

[30] Faqihi F., Alharthy A., Abdulaziz S., Balhamar A., Alomari A., AlAseri Z., Tamim H., Alqahtani S.A., Kutsogiannis D.J., Brindley P.G. (2021). Therapeutic plasma exchange in patients with life-threatening COVID-19: A randomised controlled clinical trial. Int. J. Antimicrob. Agents.

[31] Qin J., Wang G., Han D. (2022). Benefits of plasma exchange on mortality in patients with COVID-19: A systematic review and meta-analysis. Int. J. Infect. Dis..

[32] Cervantes C.E., Bloch E.M., Sperati C.J. (2023). Therapeutic Plasma Exchange: Core Curriculum 2023. Am. J. Kidney Dis..

[33] Basic-Jukic N., Kes P., Glavas-Boras S., Brunetta B., Bubic-Filipi L., Puretic Z. (2005). Complications of therapeutic plasma exchange: Experience with 4857 treatments. Ther. Apher. Dial..

[34] Kiprov D.D., Golden P., Rohe R., Smith S., Hofmann J., Hunnicutt J. (2001). Adverse reactions associated with mobile therapeutic apheresis: Analysis of 17,940 procedures. J. Clin. Apher..

[35] Couriel D., Weinstein R. (1994). Complications of therapeutic plasma exchange: A recent assessment. J. Clin. Apher..

[36] Winters J.L. (2006). Complications of donor apheresis. J. Clin. Apher..

[37] Lee G., Arepally G.M. (2012). Anticoagulation techniques in apheresis: From heparin to citrate and beyond. J. Clin. Apher..

[38] Owen H.G., Brecher M.E. (1994). Atypical reactions associated with use of angiotensin-converting enzyme inhibitors and apheresis. Transfusion.

[39] Lemiech-Mirowska E., Kiersnowska Z.M., Michałkiewicz M., Depta A., Marczak M. (2021). Nosocomial infections as one of the most important problems of healthcare system. Ann. Agric. Environ. Med..

[40] Lin Y., Zhou X., Wu J., Mei Y., Ni L., Qiu H., Zhou Y., Chen Y., Wan W. (2024). Effectiveness of double-filtration plasmapheresis in reducing immunoglobulin and culprit antibody levels in neuroimmune disorders: A single-center retrospective analysis from China. J. Neuroimmunol..

[41] Pham H.P., Staley E.M., Schwartz J. (2019). Therapeutic plasma exchange—A brief review of indications, urgency, schedule, and technical aspects. Transfus. Apher. Sci..

